# Support Stiffness Monitoring of Cylindrical Structures Using Magnetostrictive Transducers and the Torsional Mode *T*(0,1)

**DOI:** 10.3390/s18041263

**Published:** 2018-04-19

**Authors:** Jabid Quiroga Mendez, Luis Mujica, Rodolfo Villamizar, Magda Ruiz

**Affiliations:** 1 Schools of Mechanical and Electric, Electronics and Telecommunications Engineering, Universidad Industrial de Santander (UIS), Universidad Industrial de Santander (UIS), Cra 27 Calle 9, Bucaramanga, Colombia; rovillam@uis.edu.co; 2 Departament de Matemàtiques, CoDAlab, Escola d’Enginyeria de Barcelona Est (EEBE), Universitat Politècnica de Catalunya, Campus Diagonal-Besòs. C, Eduard Maristany, 6-12, St. Adrià de Besòs, 08930 Barcelona, Spain; luis.eduardo.mujica@upc.edu (L.M.); magda.ruiz@upc.edu (M.R.)

**Keywords:** guided waves, supports monitoring, torsional waves, structural health monitoring (SHM), finite elements modelling (FEM)

## Abstract

In this paper, a support stiffness monitoring scheme based on torsional guided waves for detecting loss of rigidity in a support of cylindrical structures is presented. Poor support performance in cylindrical specimens such as a pipeline setup located in a sloping terrain may produce a risky operation condition in terms of the installation integrity and the possibility of human casualties. The effects of changing the contact forces between support and the waveguide have been investigated by considering variations in the load between them. Fundamental torsional T(0,1) mode is produced and launched by a magnetostrictive collar in a pitch-catch configuration to study the support effect in the wavepacket propagation. Several scenarios are studied by emulating an abnormal condition in the support of a dedicated test bench. Numerical results revealed T(0,1) ultrasonic energy leakage in the form of $SH_{0}$ bulk waves when a mechanical coupling between the cylindrical waveguide and support is yielded. Experimental results showed that the rate of ultrasonic energy leakage depends on the magnitude of the reaction forces between pipe and support; so different levels of attenuation of T(0,1) mode will be produced with different mechanical contact conditions. Thus, it is possible to relate a measured attenuation to variations in the supports condition. Results of each scenarios are presented and discussed demonstrating the feasibility and potential of tracking of the amplitude of the T(0,1) as an indicator of abnormal conditions in simple supports.

## 1. Introduction

Pipelines in service are continuously affected by corrosion, erosion, chemical attacks, stress, extreme climate conditions, among others. Pipeline stresses have great influence in its performance during operation, affecting strength, expected operational life and dimensional stability. Stresses occurring in service, which are difficult to diagnose and identify, can unexpectedly appear and turn into invisible due to the apparent absence of an external load, such as loss of rigidity in the foundation of the pipeline supports. Besides, pipe and fluid masses may develop important normal stresses via bending moment that can compromise the integrity of the pipelines.

Supports integrity inspection presents several challenges, e.g., mechanical stress variations are produced along the pipeline when the support loads are changing. Therefore, any suitable support monitoring scheme should be focused on determining the variation of the axial stress condition in the pipe or any other feature that revels varying load conditions in the supports since load variations measurement in the support itself is difficult, impractical and often prohibitively expensive. Additionally, this abnormal condition can appear suddenly as result of terrain movements or soil weakening by variations in the soil’s water content.

Among the suitable techniques used to evaluate support integrity i.e., cracks [1], corrosion [2], and contact loads [3,4], ultrasonic-based techniques are gaining attention. The successful detection of abnormal load condition at support spots relies on the capability to discriminate the variation of the ultrasonic guided wavepacket between the support on a normal load condition (even in presence of discontinuities i.e., corrosion or notch reflected signals) and in abnormal condition (lack of rigidity foundation in the support). Consequently, it is essential to estimate changes influence in the support load condition in the guided wave; few works have been reported on this subject. The effect of clamped supports on the propagation of the torsional mode T(0,1) is studied in [3] by means of simulation and experiments. The focus of this study was the attenuation caused by the presence of rubber gaskets in the support and its consequences on the detectability of discontinuities located after the support. Moreover, a growth of the reflection produced by the support is found, as the tightness of the clamp support increase, for the T(0,1) mode. In [5,6], the interaction of the torsional mode T(0,1) with a longitudinal welded support in a pipe is modeled using FEM. Energy leakage from the pipe to the support via transmission and mode conversion are detected. The mode T(0,1) is used in [7] to evaluate the effect of a longitudinal support welded on the wavepacket propagation in a pipe using both simulation and experimental methods. It is determined that reflected signals by the longitudinal welded support are much greater than reflections from other discontinuities beyond the support.

The interaction of the fundamental torsional mode with simple supports is studied by experimental tests and analytical methods, finite elements and Semi-Analytical Finite Elements (SAFE) [4]. A SAFE analysis establishes which modes can propagate in a stress-free pipe. They concluded that exist a mode conversion from T(0,1) into a suitable mode which is propagated along that supported section. The reflection coefficient for the support will be then high where there is no mode similarity to T(0,1) in the region around the support.

As mentioned above, variations of the normal stress in pipelines can be attributed to the changes in the supports load conditions. Therefore, tracking stress variations along the pipe in a region close to the support should provide information of the support integrity. Presence of stress in the structure affects damage localization in the case of using methods that utilize elastic wave propagation. Wave propagation velocity decreases with increasing stress [8]. The study of the guided wave propagation in stressed specimens is mainly based on the acoustoelasticity effect (stress dependence of acoustic bulk wave velocities i.e., shear and longitudinal velocities). The foundation of the acoustoelasticity theory can be endorsed to the Murnaghan [9] and Hughes and Kelly [10] works in the middle part of the last century. Since, part of the research has been aimed to the determination of the acoustoelastic guided wave dispersion curves [11,12,13]; others are devoted to determine the load condition or the residual stress of the specimen based on the velocity change of the guided waves [14,15,16,17,18]. On the other hand, an investigation of wave propagation in double cylindrical rods based on Hertz contact theory and considering the effect of prestressing found that at low frequencies, group velocity of torsional-like modes are very sensitive to the variation of prestress [19]. In [20] the effect of stress applied perpendicular to the propagation-direction on guided wave propagation is accounted for through nonlinear elasticity and finite deformation theory (Acoustoelasticity). Emphasis is placed on the stress dependence of the energy velocity of the $S_{0}$ lamb modes. For this purpose, an expression for the energy velocity of leaky Lamb waves in a stressed plate is derived. The transfer of energy inside a multi-wire cable with a SAFE method has been investigated by [21]. An energy transfer parameter has been proposed in order to determine the power flow distribution inside the cable and the exchange of energy between wires. Based on this parameter, a new compressional mode excited with an axial excitation of the central wire mainly localized inside the central wire has been found. Finally, some specific engineering applications such as bolted structural connections, grouted tendons and steel stands, have been object to monitoring using the acoustoelasticity effect of guided waves [22,23,24,25]. In summary, previous research works have been focused on the study the influence of the support material changes (cracks, pinholes, corrosion, contact loads) in the guided wave propagation and how the presence of the support in the propagation path affects the detection of material discontinuities in the waveguide under investigation. The work presented here is focused specifically on formulate a methodology to monitoring changes in the load condition in the support. To do that, it is needed an understanding of how the mode T(0,1) interacts with simple supports under varying support load conditions. Experimental and numerical tests reveal a greater effect of the energy leakage instead of velocity changes as a result of the Acoustoelasticity effect.

## 2. Theoretical Background

### 2.1. Axisymmetric Torsional Guided Waves Propagating in Cylindrical Waveguides

To develop an axisymmetric wave propagation model for torsional guided waves, certain assumptions are needed. The model assumes that the cylindrical system is geometrically axisymmetric, infinitely long, stress-free in the boundaries for some specific directions and surrounded of vacuum. The material is elastic, homogeneous and isotropic (Its mechanical and thermal properties are the same in all directions). The waves will be assumed to be continuous, the frequency real (transients effects are no considered), and the energy is finite and constant. The solutions to motion equation will only be sought explored for guided waves, which are propagated axially.

**Figure 1:**
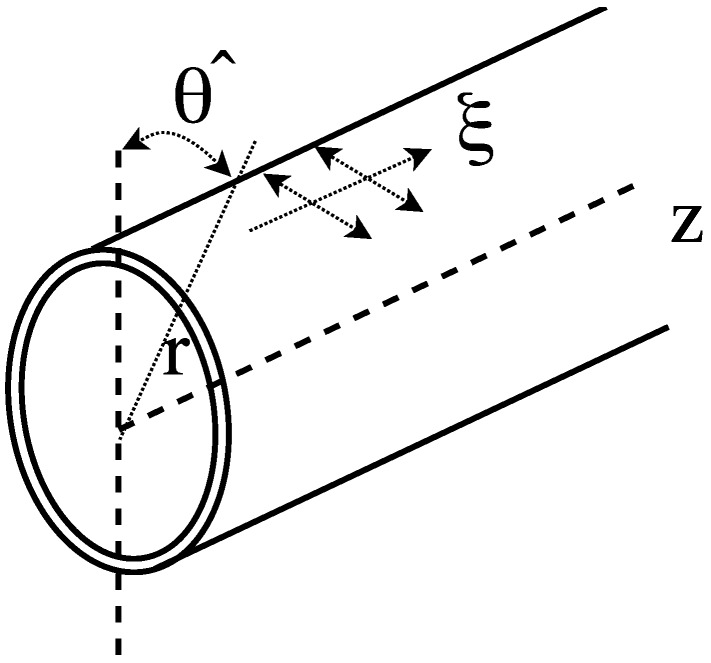
Schematic representation of cylindrical coordinates for the cylindrical waveguide.

Three guided waves modes can be developed in cylindrical systems: longitudinal (L), flexural (F) and torsional (T). Although the formers (L and F) are widely used in Structural Health Monitoring (SHM) applications, they are strongly affected by an acoustic coupling between these modes and the surroundings. On the contrary, the latter does not. The torsional modes are characterized mainly by a displacement primarily in the θ-direction. The axisymmetric torsional mode corresponds to an uniform azimuthal displacement in θ-direction (angular displacement) of the entire cylindrical waveguide; the higher order torsional modes exhibit a more complicated behavior. However, the angular displacement is not constant through the radius of the cylinder. Different locations through the radius of the cylinder can twist in different directions and nulls of displacement can exist. Figure 1 depicts cylindrical θ and *z* directions.

The guided waves propagation model is based on the combination of Euler’s equation of motion and the generalized Hooke’s law. Both relations yield the Navier’s displacement equation of motion which decoupled solution (Φ scalar field and *H* vector field) can be expressed in cylindrical coordinates Equation (1). The solution may be divided into the product of functions of each one of the spatial dimensions as:

(1) $$\Phi ,H=\Gamma_{\Phi ,H}\left(r\right)\Gamma_{\Phi ,H}\left(\theta \right)\Gamma_{\Phi ,H}\left(z\right)e^{i\left(kr-\omega t\right)},$$

where *k* is the wavenumber vector, ω is the circular frequency, *t* is the time and $\Gamma_{\Phi ,H}\left(r\right)$ , $\Gamma_{\Phi ,H}\left(\theta \right)$, and $\Gamma_{\Phi ,H}\left(z\right)$ describe the field variation in each spatial coordinate. Assuming that the wave does not propagate in the radial direction (r) and that the displacement field does not vary in the θ-direction either *z*-direction except for the harmonic oscillation described by the wavenumber as follows

(2) $$\Phi ,H=\Gamma_{\Phi ,H}\left(r\right)e^{ip\theta }e^{i\left(\xi z-\omega t\right)},$$

where ξ is the component of the complex vector wavenumber in the *z*-direction, *p* is referred to as the circumferential order; which must be a whole number, since only propagation in the direction of the axis of the cylinder is considered and the field variables must be continuous in the angular direction. Displacements and stresses can be expressed in terms of potential functions, which can be numerically solved (see [26,27,28,29] for more details on this subject).

On the other hand, the family of torsional modes results when only the $u_{\theta }$ displacement is assumed to exist $\left(u_{r},u_{z}=0\right)$. Such a displacement field is obtained only if rotational potential function in *z*, $h_{z}\neq 0$. Then, for sake of brevity, only the expressions for $u_{\theta }$, $h_{z}$, and the stress ($\sigma_{r\theta }$ as boundary condition), are used in forward to study the axisymmetric torsional modes as follows [29].

(3) $$h_{z}\left(r\right)=C_{1}J_{p}\left(\beta r\right),$$

(4) $$u_{\theta }=h_{z^{\prime }}\left(r\right)\cos\left(p\theta \right)e^{i\left(\xi z-\omega t\right)},$$

(5) $$\sigma_{r\theta }=\mu \left[-\left(2h_{z^{\prime \prime }}-\beta^{2}h_{z}\right)\right],$$

(6) $$\beta^{2}=\frac{\omega^{2}}{C_{2}^{2}}-\xi^{2},$$

where $C_{2}$ is the bulk shear velocity, $J_{p}$ is the Bessel function of the first kind an order *p*, *r* is the cylinder radius, and $C_{1}$ is a constant. The family of Bessel functions $J_{p}$ represent standing waves. For the case of the axisymmetric modes, *p*=0, and by using the property of Bessel functions $J_{0}$’ $\left(x\right)=-J_{1}\left(x\right)$, the corresponding $u_{\theta }$ can be expressed as follows.

(7) $$u_{\theta }=-\frac{\partial h_{z}}{\partial r}=\left(C_{1}\beta \right)J_{1}\left(\beta r\right)e^{i\left(\xi z-\omega t\right)}.$$

The frequency equation for the torsional modes may be obtained by using the boundary condition ${\left.\sigma_{r\theta }\right|}_{r_{ext}}=0$, for the studied case only $\sigma_{zz}\neq 0$, in this way

(8) $$\left[\beta^{2}a^{2}J_{0}\left(\beta a\right)-2\beta aJ_{1}\left(\beta a\right)\right]-\left[\beta^{2}b^{2}J_{0}\left(\beta b\right)-2\beta bJ_{1}\left(\beta b\right)\right]=0.$$

Noting that this equation belongs to the dispersion equation for the torsional waves propagating in the axial direction of a cylinder of inner and outer radius *a* and *b* respectively. The lowest axisymmetric torsional mode, the first root of Equation (8), is β=0, in which involves the rotation of each transverse section of the cylinder as a whole about its center, is not adequately described by the Bessel equations [30]. This mode corresponds to the zero order torsional mode T(0,1) and, therefore using Equation (6), and considering β=0, it is obtained that

(9) $$\omega =\xi C_{2}.$$

Considering the previous result and the phase velocity definition, let state the following relation:

(10) $$V_{p}=\frac{\omega }{\xi }=C_{2}.$$

The last expression shows that T(0,1) propagates at a constant phase velocity equivalent to the bulk shear velocity of the material. The T(0,1) mode is preferred in pipeline monitoring for two main reasons: (i) it propagates non-dispersively at the shear velocity of the medium and, (ii) since the fluid layers do not support shear waves, the T(0,1) propagates solely in the steel wall of the pipe with no energy leakage to the fluid and no attenuation. On the other hand, The zero order torsional modes can be expressed analytically in a similar fashion as the ‘SH’ modes in a plate.

### 2.2. Generation of Torsional Modes by the Magnetostrictive Principle

**Figure 2:**
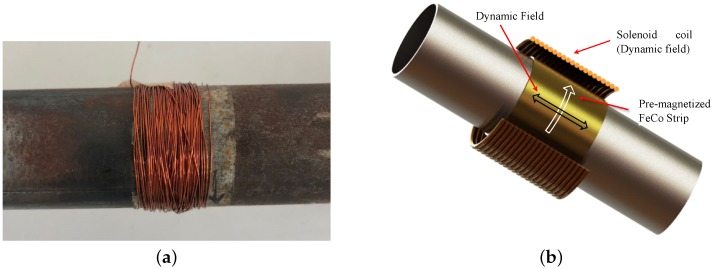
Magnetostrictive transducer used in the test bench. (**a**) Picture of the transducer and (**b**) Schematic representation of the magnetostrictive strip and the coil.

**Figure 3:**
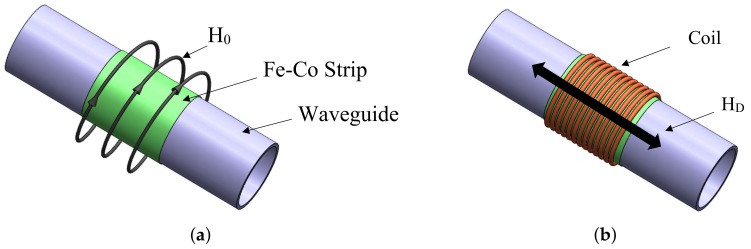
Steps for torsional guided wave generation based on magnetostriction principle. (**a**) Induced static magnetic field “$H_{0}$” in the FeCo strip and (**b**) Dynamic magnetic field “$H_{D}$” in the specimen.

In this work, the fundamental torsional mode is generated and captured by using the magnetostriction principle. One advantage of this cost-effective method of transduction is the fact that coupland is not required between the specimen and the transducer [31]. On the other hand, the magnetostriction principle provides better performance in varying temperature environments compared to the piezoelectricity-based transducer, mainly for the absence of a couplant. In the literature, it is reported that the temperature influence in the propagation of guided waves for a range (from 20–200 °C) generates a drop in amplitude (9 db) [32]. The basic configuration of the transducer is illustrated in Figure 2b. The set is comprised of two parts. The first one is a solenoid coil which provides a dynamic magnetic field to the pipe or measures the induced magnetic field through the propagation of the torsional guided wave in the waveguide. The second one, is a magnetostrictive strip which supplies a residual circumferential bias static field. The residual magnetization of the strip is obtained rubbing it with a magnet in the circumferential direction. Thus, the most critical component of a magnetostrictive system is the magnetostrictive material (ferromagnetic strip) used to transform electrical energy into mechanical and vice versa. Its transduction efficiency in both acquisition and transmission mode depends mainly on its physical properties. As a consequence, these physical parameters must be considered to select the right ferromagnetic material to be used in magnetostrictive transducers. For this reason, a magnetostrictive probe was acquired from Guided Wave Analysis LLC company. The MsS probe consists of amorphous cobalt–iron alloy strips (0.15 mm thick), which have lower eddy current loss and larger magnetostriction [31]. The solenoid coil was constructed of enameled wire of 0.32 mm of diameter (28 aws) copper wound around the strip with 50 turns along its width, a system with an Arbitrary Wave Generator (AWG) and amplifiers was used to excite the coil with the modulated pulse. In general, to create a torsional stress, a helical magnetization is induced in the ferromagnetic strip through the following procedure:

1. A bias magnetization ($H_{0}$), static magnetic field, in the strip is induced in circumferential direction through the passage of a permanent magnet as shown in Figure 3a.
2. A second alternate magnetic field $H_{D}$ of lower magnitude than $H_{0}$ is created in axial direction by the current flowing into a coil that surrounds the pipe circumference at the strip position as seen in Figure 3b.
3. Finally, the combined magnetic fields generate an applied alternate helical field that according to the Villari effect will create torsional vibrations via shear stress.

### 2.3. Single Mode Generation of T(0,1)

A key element of the inspection system is the selection and exploitation of a single mode. In general, an excitation source can excite all of the modes which exist within its frequency bandwidth. Therefore, although troublesome to achieve, it is essential to design the transducers and the signal to excite only the chosen mode. In the experiments covered by this work, the chosen mode for excitation in the inspection system is T(0,1). This mode is very attractive because is non-dispersive over a wide bandwidth, so the signal shape and amplitude are retained as it travels. The T(0,1) mode consists of an axisymmetric torsion of the pipe and it can only be produced by shearing stresses (circumferential motion). In order to excite only this mode, some aspects must be considered.

- At low excitation frequency, the possibility to generate longitudinal and flexural modes is reduced.
- As the flexural modes are not axisymmetric, they can be avoided when the transducer elements are arranged axisymmetrically.
- Excitation frequency must be chosen below or equal to 50 kHz because the cutoff frequencies of the lowest longitudinal and flexural modes in the dispersion phase velocity curve are above.
- Due to that the dynamic magnetic field imposed over the pipe is smaller than the bias magnetic field, it is not expected a meaningful magnitude of longitudinal modes.
- The width coil should be small or equal to half the wavelength of the target wave mode at an excitation frequency; otherwise, the vibration caused by the wire at the end of the coil will offset each other, reducing the vibration intensity of the expected guided wave mode [33].

Based on the previous considerations and the recommendations of the magnetostrictive transducer provider, an excitation frequency of 32 kHz was chosen. For this frequency a wavelength of 0.1 m is calculated. Thus, according to the provider the strip has to be smaller than a 1/3 of the wavelength at the excitation frequency. Therefore, a strip and coil of 2 cm of width are used.

## 3. Experimental Setup

Variations in the support’s loading will produce an increment in the normal stress along the pipe. Therefore, tracking the axial stress could provide an indication of abnormal loading condition in the support. Thus, a test bench is implemented to emulate a failure or a change in the loading support conditions. In the proposed methodology, a scheme of two magnetostrictive collars in a pitch-catch configuration is adopted to produce the T(0,1) torsional mode.

**Figure 4:**
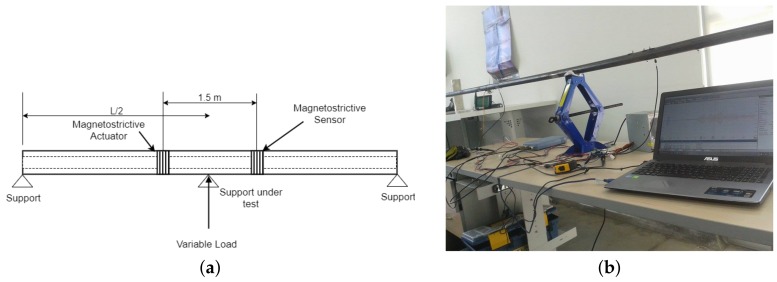
Implemented test bench to emulate different support stiffness. (**a**) Schematic representation of the hollow cylinder test bench and (**b**) Picture of the actual test bench.

**Figure 5:**
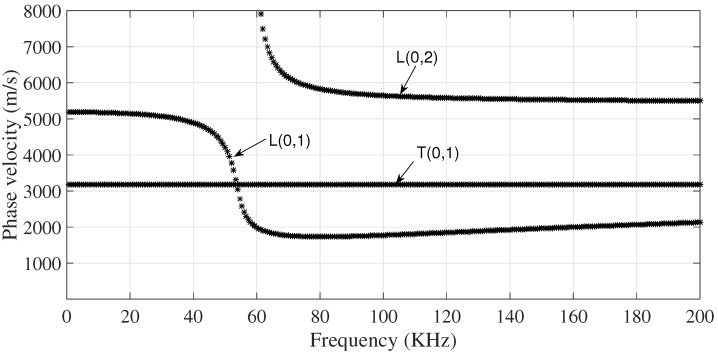
Torsional and longitudinal axisymmetric modes phase velocities for a homogeneous isotropic hollow cylinder (A-106, 1${}^{\prime }$${}^{\prime }$, Sch 40).

Experimental tests were performed on a test bench, which represents a scaled mimic of a current installation. A 1${}^{\prime }$${}^{\prime }$, L = 6 m of length, schedule 40 (external diameter = 33.4 mm and thickness = 3.3 mm), A-106 pipe supported at the free ends by fixed support, where the different stressed scenarios are produced by changing the magnitude of the reaction in the variable support located in the middle of the pipe (L/2), as shown in Figure 4. The phase velocity dispersion curves of the axisymmetric modes of 1′′, A-106 pipe are presented in Figure 5.

**Figure 6:**
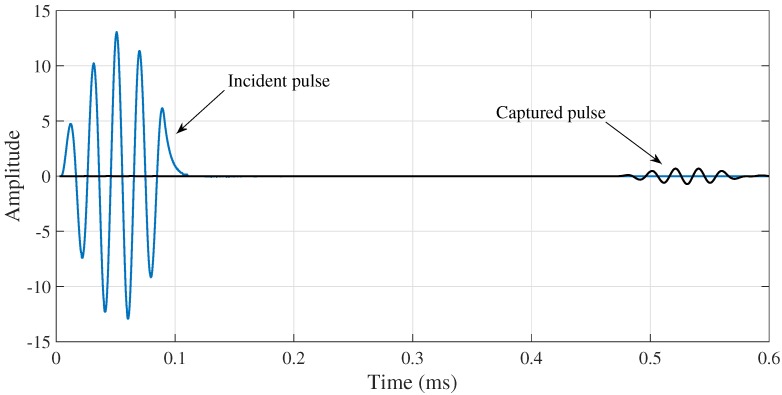
Experimental incident and captured T(0,1) pulses at excitation frequency of 32 Khz in a 1${}^{\prime }$${}^{\prime }$ A-106 Sch. 40 pipe.

The waveguides are excited with a sinusoidal pulse of 5 cycles Gaussian-modulated at 32 kHz via magnetostrictive actuator (see Figure 6). The magnetostrictive actuator is composed by two elements wrapped around the pipe; a thin FeCo alloy strip (adhered to the pipe by epoxy) to yield a circumferential magnetic field and, an electromagnetic coil with an alternate current flowing in circumferential direction used to produce an axial magnetic field. The combined action produces a helicoidally strain pulse along the pipe. Therefore, torsional guided waves are generated by axisymmetric surface loading. In order to launch only the T(0,1) mode, an appropriate selection of the excitation frequency and wide of the strip must be performed (See Figure 2). The torsional guided wave propagates along the pipe and it is captured 1.5 m. ahead of the actuator with a magnetostrictive sensor. As it is expected, the velocity of propagation was very close to the shear velocity for the material of the pipe 3200 m/s). No other modes were detected in the tests. A picoscope 2208 is used as DAQ system.

**Figure 7:**
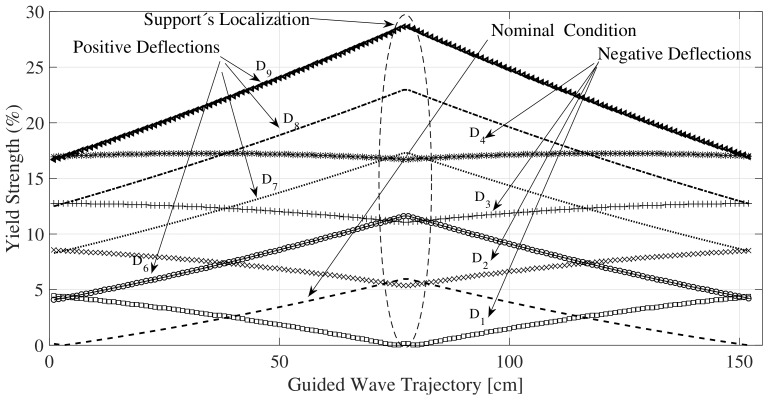
Stress variation along the pipe subject to different reaction forces in the support.

The experiments were conducted in such a way that it replicates a common real pipe under a simplified loading and support conditions. Variations in the stiffness are emulated by changing the magnitude of the variable support. First the nominal condition is determined considering the absence of deflection with respect to the vertical (*g*-direction) in the middle variable support. Under this condition the middle part of the pipe (L/2) is experimenting a negative bending moment and the pipe develops an internal stress of around 5% of the yield strength, under this scenario the load in Newtons between support and pipe is 92.2 N (reference stiffness). The magnitude of the load applied for the variable support to the pipe is decreasing for $D_{1}-D_{4}$ scenarios for deflections in gravity direction and is increasing for $D_{6}-D_{9}$ in opposite direction. In this way, $D_{1}$ belongs to a deflection of 10 mm downwards (74.1 N), $D_{2}$ to 20 mm (57.03 N), $D_{3}$ to 30 mm (39.46 N) and $D_{4}$ to 40 mm (21.9 N). Similarly, $D_{6}$ belongs to 10 mm upwards (109.75 N), $D_{7}$ to 20 mm (127.3 N), $D_{8}$ to 30 mm (143.9 N) and $D_{9}$ to 40 mm (160 N). The test are configured in such way that the stresses correspond to an incipient strength condition (<30% of yields strength). The variation of the deflection yields an increase in the magnitude of the bending moment in the middle part of the pipeline, as shown in Figure 7.

Figure 7 provides an estimation of the normal stresses along the pipe (z-direction), expressed in terms of percentage of yield strength, generated by bending. The maximum bending stress in a cross-section for a specific position along the pipe axis is located at the outer distance (the exterior radius) which means the pipe’s surface is the area under highest stress.

**Figure 8:**
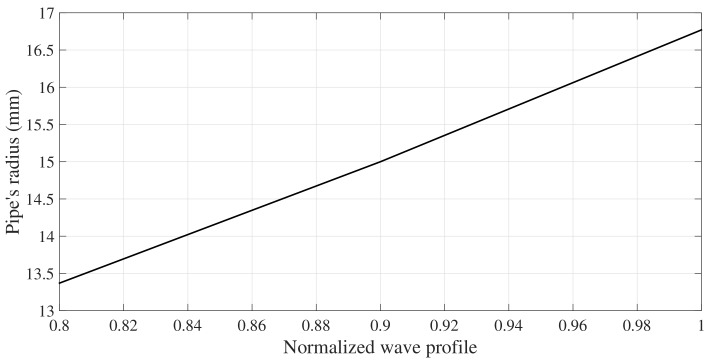
Sample wave structure of $U_{\theta }$ for T(0,1) at 32 kHz in a 1${}^{\prime }$${}^{\prime }$ sch. 40 pipe.

Therefore, as shown in Figure 7, changes in the supports reaction will produce a distributed normal stress response along the path of propagation. In fact, the longitudinal normal stress varies from point to point along the pipe’s axis, and linearly with the radius, passing from tension to compression in the neutral axis or cylinders center; reaching a maximum normal stress value at the outmost distance from neutral axis or external radio. As shown in Figure 8, the wave structure obtained by using the GUIGUW software [34], the maximum azimuthal displacement component of the torsional wave expressed in the wave structure matches the maximum azimuthal axial stress obtained in pipes cross-section. Changes from tension to compression in a specific cross section in the cylinder must produce variations of phase velocity of T(0,1) in the same plane. Therefore, different phase velocities around $V_{0}$ for the same cross-section may result when the torsional mode is propagating in a bending pipe. On the other hand, variations in the guided waves velocities can also be attributed to a thickness reduction of the waveguide produced by corrosion or erosion. Nevertheless, these discontinuities slowly appear in the waveguide in a much greater time scale compared to the most probable scenario of unexpected change in the supports reaction. Therefore, any suddenly change in the TOF of the wave can be interpreted as a modification in the stress condition in the waveguide.

## 4. Experimental Results

**Figure 9:**
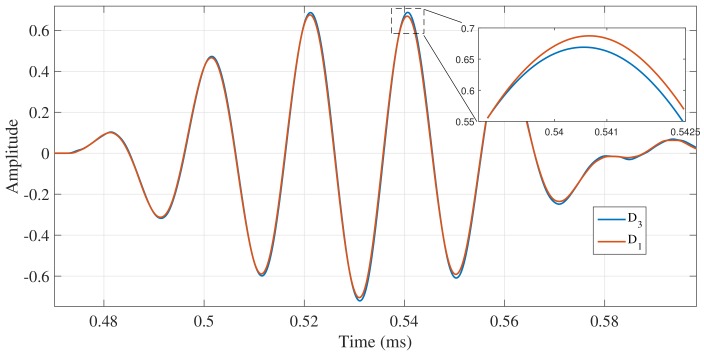
Captured fundamental torsional signals for D3 and D1.

This section provides the results of the experiments carried out under the conditions described above. One hundred tests were conducted in each scenario and the propagated torsional wave were captured. As an example, in Figure 9 one of the captured signals for the scenarios $D_{3}$ and $D_{1}$ is shown.

On the other hand, when considering the acoustoelasticity effect, variations in the pipe’s stress are not enough to produce any significant change in the TOF of the torsional guided wave under the test experimental conditions, not even the material elongation effect is observed. Considering the DAQ sampling frequency (18 MHz), the frequency of the signal (32 KHz) and the velocity of the torsional wave (around 3200 m/s), the minimal change of velocity that can be detected is 0.38 m/s (without considering noise), which represent a capability to detect phase velocity variations around 0.012%. The inability to detect $\Delta \left(TOF\right)$ may be attributed to the phase velocity in a specific pipe cross-section for a bending pipe is radius dependent due to the linear stress distribution in *r*, going from tension to compression or vice versa at the same cross section, i.e., the magnetostrictive sensor is capturing at the same time, torsional waves propagating at different phase velocities, in the case of tension stresses $V_{\sigma }<V_{0}$ and $V_{\sigma }>V_{0}$ for compression stresses.

On the other hand, as it is expected, no dispersion is observed in the captured torsional wave. Therefore, only amplitude changes are observed in the wavepackets when they are compared in time domain. The variations in the wave amplitude, which can be interpreted as energy variations, could be attributed to the fact that the interaction between the torsional guided wave and the support has been altered. Although the experimental tests reveals tiny amplitude changes in the captured signal, the abnormal condition to be monitored, a decrease of the support stiffness, produces an anomalous behavior in the guided wave, i.e., an increase of the transmitted pulse magnitude.

In order to create a feature associated with the amplitude variation of the captured signal as a result of the acoustic coupling between pipe and support, an auto correlation of the incident signal, $r_{xx}$, and a cross correlation between the current sensed signal $S_{1}$(*t*) and the incident signals $S_{0}$(*t*), $r_{xy}$, are used instead of the raw signals to produce a relation between both signals. Autocorrelation and cross correlation are calculated as follows:

(11) $$r_{xx}\left(n\right)=\frac{1}{N}\sum_{n=0}^{N}S_{0}\left(n\right)S_{0}\left(n-t\right),$$

(12) $$r_{xy}\left(n\right)=\frac{1}{N}\sum_{n=0}^{N}S_{0}\left(n\right)S_{i}\left(n-t\right),$$

where *N* is the number of signal samples, *t* is defined in the interval $\left(-N*T_{s},\left(N-1\right)*T_{s}\right)$. Then, the Root Mean Square Deviation (RMSD) is calculated as:

(13) $$RMSD=\sqrt{\frac{\sum_{i=1}^{N}{\left(G_{i}-ref\right)}^{2}}{N}},$$

where $G_{i}$ is a scalar calculated using the peak values of the correlated signals:

(14) $$G_{i}=\frac{max(r_{xy})}{max(r_{xx})}.$$

**Figure 10:**
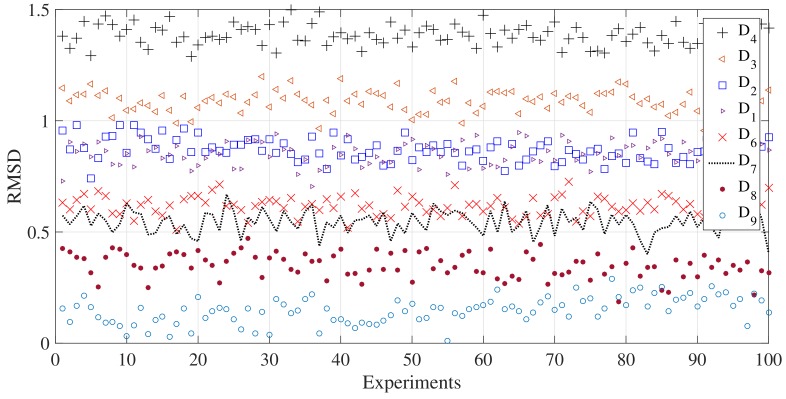
RMSD for the studied scenarios.

**Figure 11:**
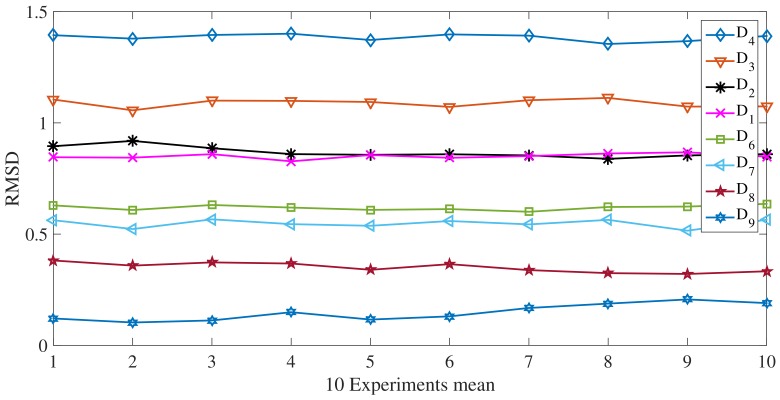
Experimental Ten-averaged RMSD values for the studied cases.

**Figure 12:**
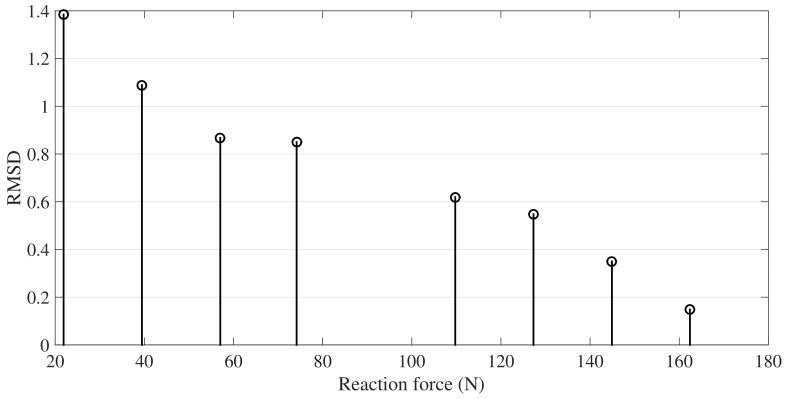
Experimental results of RMSD versus Reaction force between pipe and support.

$G_{i}$ denotes the peak value of the current scenario, ref is the peak value of the averaged signal (100 experiments) at nominal condition, (no deflection in the pipe at the center). Since only magnitude changes are expected in this monitoring scheme, RMSD is proposed to evaluate the supports rigidity. In Figure 10, it can be seen the RMSD values for the different scenarios. Now, ten runs were averaged and presented in Figure 11 and finally, Figure 12 presents the relation between force and RMSD for the different cases.

Although $D_{6}$ to $D_{9}$ are improbable scenarios in a real installation, deflection opposite to the gravity, here, they are used to determine in a wide perspective how the torsional guided wave interacts with the reaction force exerted by the simple support. Variations in RMSD are the result of change in the contact conditions or the magnitude of the supports force. Clearly, as the magnitude of the supports reaction decrease, the amplitude of the wave captured by the sensor increase. This result can be explained from an energy perspective, in which, a reduction of the force between the pipe and the support produce a decrease of the transmitted energy to the supports material and therefore, a greater wave refraction.

## 5. Finite Element Modeling Analysis

Considering the previous results—a very small acoustoelastic effect and a change in the amplitude of the wave—in the following numerical analysis, we investigate the influence of the interaction between support and the waveguide in the T(0,1) propagation by means of FEM simulations. This analysis has been restricted to the transmission of ultrasound energy to the support. Thus, the FEM does not pretend the estimate the actual change of the magnitude of the transmitted pulse, its only aim is to observe the relation between reaction force and $SH_{0}$ generation. A mode conversion of T(0,1) transmitted through the support, modeled as a plate, is expected because the tangential nature of the T(0,1) and the mechanical coupling between the pipe (cylinder) and the simple support (plate).

Since surfaces between pipe and support are rough, a contact interface can be assumed as a series of parts in contact and voids. Due to the high acoustic impedance of the air, voids act as reflectors for the ultrasonic waves. Thus, an increase of force will result in a reduction of the number and size of these voids and, at the same time, an increase of the ultrasound energy transmitted through the interface. When two surfaces of the same material are in contact, there are no variations of acoustic impedance in the contact area created by the matching micro-asperities generating the absence of material discontinuities for the transmission of the ultrasonic waves [35]. In numerical analysis, a real contact in metallic interfaces (no perfect) is not an easy task to model, a simple version of the contact area is implemented in the performed simulations.

**Figure 13:**
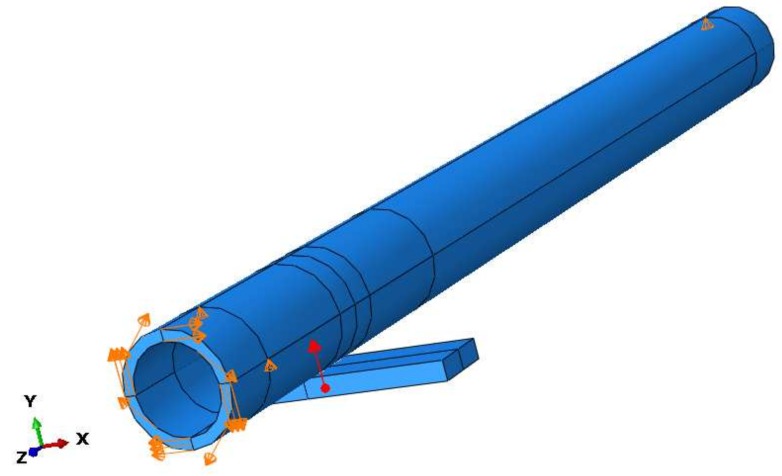
Schematic representation of the simulated pipe with a simple support.

A 3D FEM model was built representing a scaled but equivalent version of the experimental setup to reduce the size of the FEM model and consequently the computational cost. The steel pipe of 1 inch schedule 40 (outer diameter: 33.4 mm and wall thickness: 3.38 mm) is modeled as a hollow cylinder with an axial length of 0.4 m. The simple support is represented by a steel plate of 6 mm of thickness and with the same material as the pipe, two boundary conditions in the cylinder constrain the displacement in y-direction of both extremes of the cylinder. Changes in support stiffness are configured varying the magnitude of a vertical concentrated force in upward direction situated in the middle part of the plate as shown in Figure 13.

The material properties used for steel were assumed as follows: Density ρ = 7830 kg/m${}^{3}$, Young’s modulus (*E*) = 210 GPa and Poisson’s ratio ν = 0.3. To ensure an adequate mesh refinement level, the minimum allowed inter-nodal length $L_{min}$ is calculated. The lowest phase velocity $C_{T}$ (i.e., transverse or shear wave speed), and consequently the shortest wavelength establishes the minimum permissible mesh size so spatial aliasing due to the finite element discretization does not occur [36]. Considering the frequency and the steel shear wave velocity, $L_{min}$ is calculated as follows:

(15) $$L_{min}=\frac{C_{T}}{n_{min}f_{max}}=\frac{\lambda_{min}}{n_{min}},$$

where, $n_{min}$ is the number of elements across the smallest wavelength of interest (assumed in this case as $n_{min}\geq 10$) [37], and $f_{max}$ the maximum frequency of interest. Considering $n_{min}=15$, $f_{max}=3200$ Hz and $C_{T}=3200$ m/s, the minimum element length results, approximately 6.66 mm. Therefore, seeds size of 2 mm can be considered as a sufficient mesh refinement. In addition, for time domain models solved with an explicit scheme, an adequate integration time step Δt assures a more accurate solution. In general, simulation accuracy can be increased with increasingly smaller integration time steps but punished by a higher computational cost. So, the time step Δt has to be smaller than the critical time step $\Delta t_{cr}$ which is the transit time of a dilatational wave through the smallest element in the model can be calculated as follows [38]:

(16) $$\Delta t\leq \Delta t_{cr}=\frac{L_{min}}{C_{L}},$$

where $C_{L}$ is the velocity of the dilatational wave. A Δt of 5ns meets these criterion (Considering $L_{min}=2\;mm$ and $C_{L}=5944\;m/s$) and it is used to solve the model. A total of 9980 linear eight node brick element (C3D8) has been used with 52 elements around the circumferential section of the pipe.

The torsional wave is produced by a shear load at the left end face of the cylinder by a 5 cycles Hanning-window tone burst of 32 kHz. The model is configured such as the torsional wave freely propagates along the *z*-axis for 50 mm until a contact with the simple support. A 12 mm contact line is established between the pipe and the support. The whole length of the pipe model is 0.2 m with a mesh of 9980 C3D8 elements and 104 semi-infinite CIN3D8 elements. In Figure 13, a schematic representation of the simulated geometry is presented. The latter elements are used at the cylinder right face to avoid reflections (absorbing boundary) [37]. The torsional wave propagates continuously into the absorption elements without any reflection.

A key aspect of the problem at hand is the surface contact FEM modeling between the pipe and simple support. This part of the modeling must be exhaustively considered in order to derive the most realistic acoustic coupling. Nevertheless, it is worth noting that though the connection between applied force and the transmission of ultrasonic waves from the interface is qualitatively known, it is not so easy to establish a general quantitative relationship, since too many parameters (surface roughness, type of material and frequency of incident wave) influence the phenomena. The acoustic interaction between two bodies in mechanical contact is a relatively complex model, therefore several simplifying assumptions are made: The Contact between the two surfaces is assumed to have smooth surfaces, applied normal stress within the elastic limits of their materials and contact surface be non-conformable. Now, Considering a portion of pipeline lying on a simple support, represented as a plate, such as that shown in Figure 13. A idealized contact interface (line) occurs between the two specimens with an extent *l* along the axial direction of the pipe, a width *w* along the pipe perimeter. In Hertzian contact theory, two stiffness must be defined, a normal stiffness $S_{n}$ that acts in the radial direction, and a tangential stiffness $S_{t}$ that acts in the θ and *z* directions. In order to simplify the use of the Hertzian contact theory the assumption of non-conformable contact between the two bodies is assumed, i.e., both surface keep their shape around the contact zone during the contact. In practice, this assumption is not realistic because under loading, the plate may curve around the pipe, or the pipe may flatten around the contact zone depending on their stiffness. In such case, the contact model increases its degree of complexity since the contact stiffness can vary considerably along the interface. This complex model is beyond to the scope of the study of the acoustic coupling between the fundamental torsional mode T(0,1) and the $SH_{0}$ mode of a plate Because the contact is assumed to occur in a line, infinitely long cylinders with parallel axes come into contact; away from end effects, the (2D) Hertzian theory must be used. Hertzian approach provides the width in the interface of the contact area when a normal load is applied to the contact surface. Assuming Young’s modulus and Poisson ratio is close enough in magnitude between the two bodies in contact. The following expression is used to determine the width of the contact area:

(17) $$w=\sqrt{\frac{8FR^{*}}{eE^{*}}},$$

where *F*, also referred as contact loading, is the total normal force by the two bodies are pressed together along the interface length *e*, $E^{*}$ is the contact module and $R^{*}$ is the relative radius between the two bodies, determined by:

(18) $$\frac{1}{R^{*}}=\frac{1}{R_{1}}+\frac{1}{R_{2}}.$$

Note, Equation (18) is originally expressed for an interface between two cylinders, where $R_{1}$ and $R_{2}$ are, respectively, the radius of the first and second cylinder. Notice, for the studied case, the contact interface is produced by a cylinder (pipe) of exterior radius $R_{1}$, and a plate with $R_{2}$ tends to infinity. Therefore, The relative radius $R^{*}$ is $R_{1}$. The contact modulus $E^{*}$ for the same material is given by

(19) $$E^{*}=\frac{E}{2\left(1-\nu^{2}\right)}$$

The interface width obtained by Equation (17) to simulate contact between the 1${}^{\prime }$${}^{\prime }$ pipe and the plate of (6 × 9 × 20 mm), result be smaller than the gap between nodes (mesh size) used for the guided wave simulation. Therefore, the interface width is assumed as a line of shared nodes between the cylinder and the plate.

Contact treatment is internally represented by linear springs between the slave nodes (plate) and the nearest master segments (pipe). The stiffness of these springs determines the force that will be applied to the slave nodes and the master nodes. The mathematical model chosen to enforce the contact compatibility is the penalty method which requires the estimation of normal and tangential contact stiffness. In [39], it is reported that normal interface stiffness $S_{n}$ for contact between two bodies of identical material properties is given by:

(20) $$S_{n}=\frac{\pi e}{2\left(\frac{1-\nu^{2}}{E}\right)d^{*}-\frac{1}{E^{*}}},$$

where,

(21) $$d^{*}=2\ln\left(\frac{4d}{w}\right)-1,$$

$d=2R_{1}$. Although there is a lack of knowledge with respect to the small-scale properties and the small-scale dynamics of the contact interface, one of the most convenient ways to establish a realistic value for the tangential stiffness of a contact interface is to use empirical models such as the one for contact between rough surface using ultrasonic method developed by [40]

(22) $$\frac{S_{t}}{S_{n}}=\frac{2(1-\nu )}{2-\nu }$$

Equation (22) describes a constant ratio between tangential and normal stiffness. It is acknowledged that there is not an effective analytical method to determine contact properties when the bodies in contact are real. Therefore, here it is assumed values close to the obtained by the expressions cited above and checking the result against experimental evidence.

**Figure 14:**
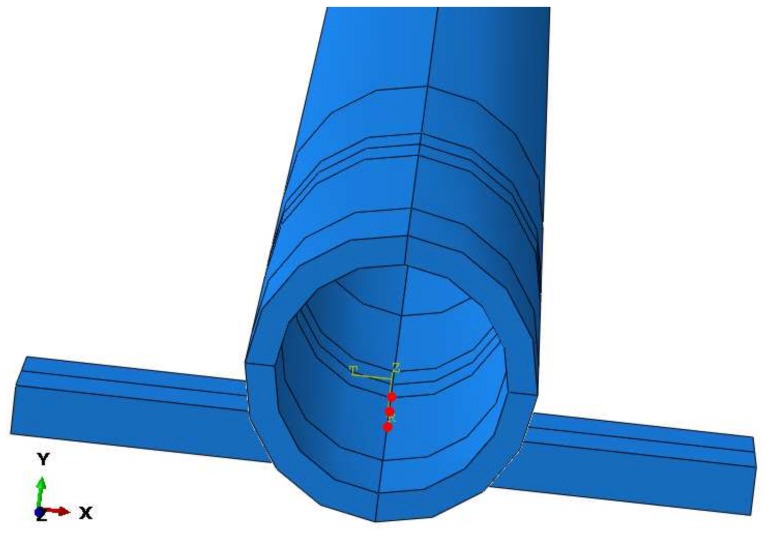
Coupling nodes in the simulated contact interface.

Some details of the contact implementation are the following: The interface between cylinder and plate is performed by a surface-surface contact and their interaction is achieved using a dynamic coupling between the nodes belonging to the contact line as presented in Figure 14. Discretization of the contact area into elementary units (contact nodes) is responsible for the contact stress (strain) acoustic transmission of the guided wave from pipe to support. The node to node discretization is appropriate for ultrasonic transmission problems because this method is suitable for small deformations and small slip. Although both materials are modeled with the same mechanical properties, the pipe is assumed as master surface and the support as slave. Besides, a linear behavior of the Pressure-Overclosure relation is assumed.

The Finite Element Analysis (FEA) has been performed to study the effect of the supports stiffness (plate) in the transmitting potential of ultrasound energy through the contact interface between the cylinder and plate. T(0,1) mode primarily has a tangential displacement $U_{\theta }$ which is transmitted by coupling to the plate. As previously mentioned, the frequency spectrum of T(0,1) has the same shape as for SH waves in a plate. In fact, the torsional modes for a cylindrical waveguide are considered the analogue of the SH plate modes [29]. Thus, for the studied case is highly likely the generation of SH waves in the support.

**Figure 15:**
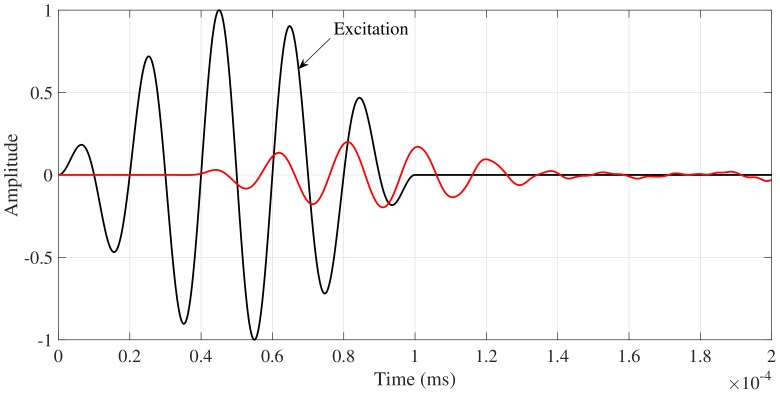
Numerical results of T(0,1) mode captured at 0.12 m of the excitation surface and at 0.07 m of the interaction point between support and pipe for the nominal condition.

**Figure 16:**
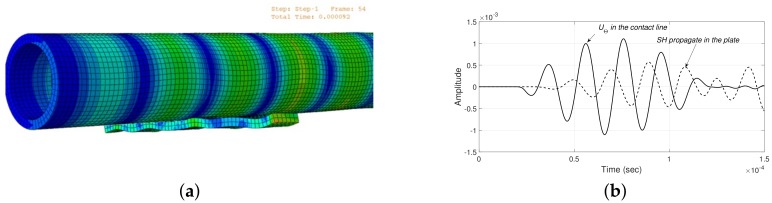
Numerical results of interaction between T(0,1) and the simple support. (**a**) Snapshots of T(0,1) mode propagation in a pipe with a simple support and (**b**) $SH_{0}$ generated in the plate for mode conversion of T(0,1).

The excitation and the fundamental torsional mode T(0,1) captured by the nodes that represent the sensor in the model are illustrated in Figure 15. The FEM simulation results of the interaction between cylinder and plate when a torsional guided wave T(0,1) mode is propagating is presented in Figure 16a. This Figure reveals that when the T(0,1) mode impinges on the support, some portion of the ultrasound energy is transmitted to the plate producing SH lamb waves.

In Figure 16b, it is depicted the generated wavepacket in the plate, it is identified as a $SH_{0}$ mode by using three different tests. First, the calculation of the phase velocity of the generated pulse belongs to velocity of the $SH_{0}$ mode reported in the dispersion curves. Second, although in Figure 16b, the generated pulse seems dispersive, variations in the wave pattern are attributed to plate edge reflections. Shorter time period FEM simulations, (not showed here) reveals a non-dispersive behavior. Lastly, after several simulations by using different plate thickness, in all cases the magnitude of the $SH_{0}$ phase velocity is constant and equal to the velocity of the shear wave. Therefore, $SH_{0}$ phase velocity is independent to plate thickness.

**Figure 17:**
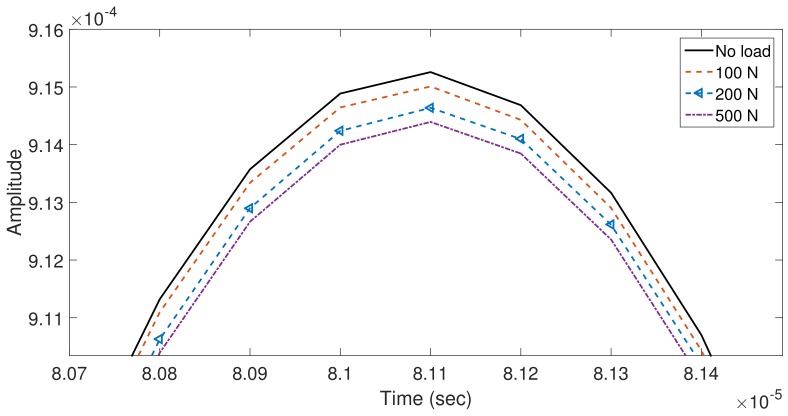
Zoom view of the wavepackets highest peaks simulated for different loads applied in the plate.

On the other hand, as long as the contact force between pipe and support is increasing, a higher ultrasonic energy is transmitted to the plate in the shape of $SH_{0}$ waves. This fact is revealed in Figure 17; where it is noted an amplitude reduction in the captured pulse for a node which it is set up as a sensor in the model; for loads which emulate different support stiffness i.e., (0, 100, 200 and 500 N). Although in Figure 17 the zoom view correspond to the highest peak, the described behavior, decrease of the wave magnitude, is presented along the simulated T(0,1). Therefore, a reduction of the reaction force between the pipe and the support by a loss of rigidity in the support foundation produces an increase with respect to a pre-defined stiffness in the magnitude of the T(0,1) captured pulse located ahead the support contact area.

## 6. Conclusions

The effects of supports external loading, stiffness, on the propagation of torsional guided wave mode T(0,1) was investigated. Experimental tests were conducted for various magnitudes of loading in a one-inch sch 40 pipe. Experimental results showed an unnoticeable influence of pipe normal stress levels, generated by bending, in the velocity of the T(0,1) mode as a consequence of the acoustoelasticity effect. No change in the TOF were reported among the different stress levels under loading varying reactions in the supports. FEM simulations of the effect of the mechanical contact between pipe and support in the propagation of T(0,1) revel a mode conversion in the interface from T(0,1) propagating in the pipe to $SH_{0}$ generated in the support, modeled as a plate. The leakage is identified comparing the magnitude of T(0,1) propagating with and without mechanical contact with the support. No damping is considered in both conditions. Experimental results exposed amplitude change of the captured torsional guided wave for different stiffness in the supports. Although the setup is plenty of symmetry (geometry of the waveguide, in loads, in the guided wave propagated, in the energy and stress distribution in the cross-section) the only asymmetric effect was the variations in the amplitude of the captured torsional wave. It is noted an attenuation growth in the transmitted wave for an increment of the supports reaction force, independent of the shape adopted by the pipe, i.e., concave upwards and concave downwards. Additionally, scattering, variations in the thickness, notches are absent in the experimentation performed. Based on these, it can be concluded that the mechanism responsible of the variations of the magnitude in mode T(0,1) transmitted in a pipe in mechanical contact with a support is the transmitted ultrasonic energy for the guided wave to the support via mode conversion from T(0,1) to $SH_{0}$ in the support. On the other hand, the presence of geometrical and material acoustic discontinuities in the guided waves such as notches, changes in the dimensions of the members cross section, corrosion or erosion, produce a reduction of the transmitted energy due to the interaction of the wave with the discontinuity (scattering, mode conversion, reflection). For all the cases, the energy reflection and mode conversion will produce a decrease in magnitude in the wave transmitted past the discontinuity. Therefore, any ultrasound wavepacket magnitude increment for a pitch-catch configuration located in the path of a support with nonexistence of an extra ultrasound source must be unequivocally considered as a loss of rigidity in the support. Besides, the material discontinuities appear in the waveguide in a much greater time scale compared to the most probable scenario of unexpected change in the supports reaction. Therefore, it can be proposed a dedicated supports stiffness monitoring scheme which basis is the tracking of short time amplitude increments of T(0,1) mode propagated in the path of the support.
